# Supplementary material for: Matrix‐Rigidity Cooperates With Biochemical Cues in M2 Macrophage Activation Through Increased Nuclear Deformation and Chromatin Accessibility
Source: Adv Sci (Weinh). 2025 Jan 19;12(8):2403409. doi: 10.1002/advs.202403409 (PMC11848612; doi:10.1002/advs.202403409)
Supplement: Supplementary file 1 — Supporting Information [file ADVS-12-2403409-s002.docx]

**
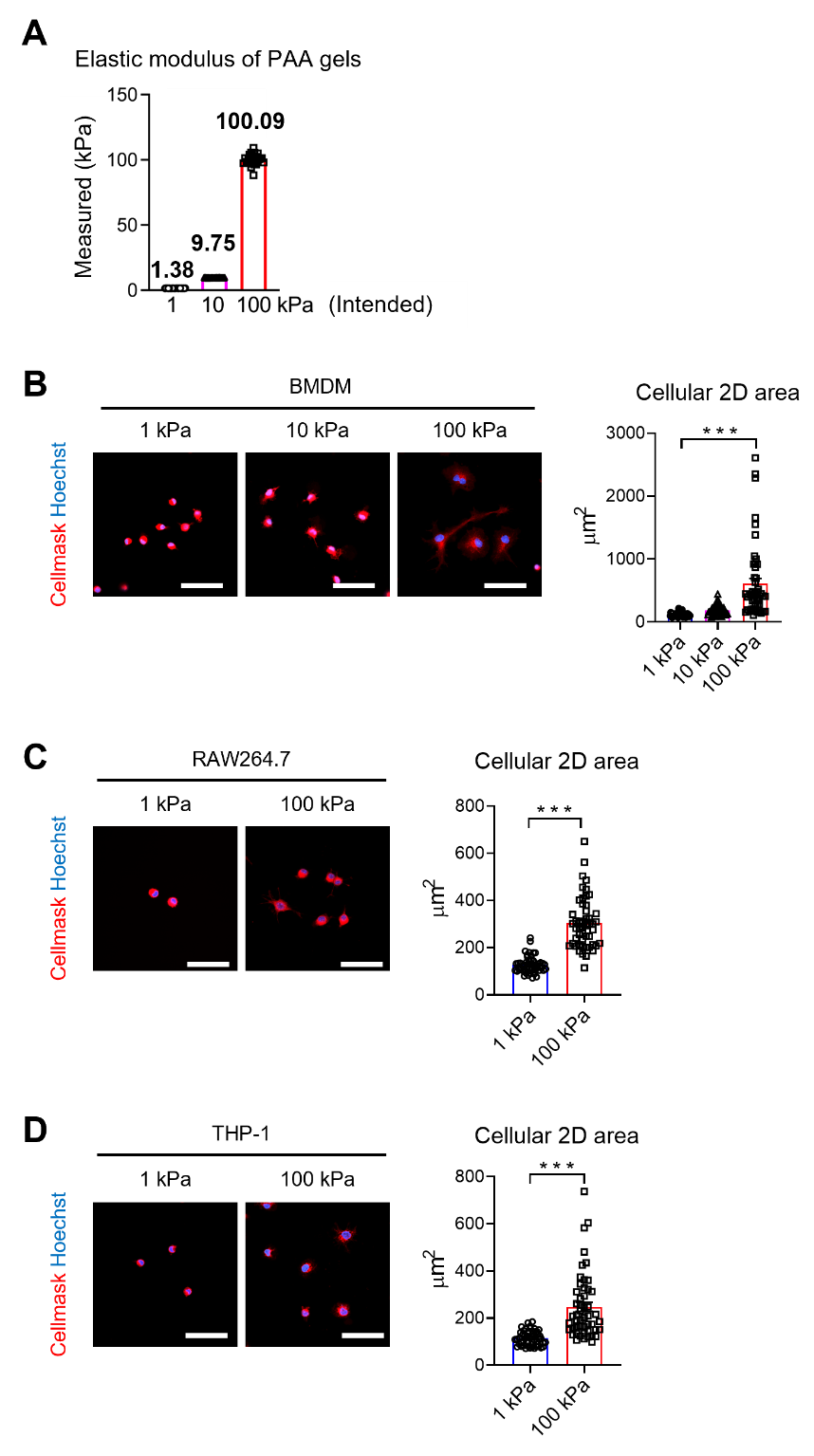
**

**Fig. S1. Macrophages undergo morphological changes in response to varying matrix stiffness.** **(A)** Stiffness of polyacrylamide (PA) gels, assessed by a nanoindentation method, resulting in 1.38 (± 0.039), 9.75 (± 0.126), and 100.09 (± 4.23) kPa from 25 measurements each, which closely align with the intended values of 1, 10, and 100 kPa, respectively. **(B-D)** Representative images of CellMask (red) and Hoechst-33342 (blue)-stained BMDMs **(B)**, RAW264.7 **(C)**, and THP-1 cells **(D)** cultured on PA gels with varying matrix rigidity (1, 10, or 100 kPa). Scale bars represent a length of 50 µm. Quantification of cellular 2D projection area is presented as mean ± SEM (*n* = 30 cells/group; ****p* < 0.001; Kruskal-Wallis test or Mann-Whitney test).


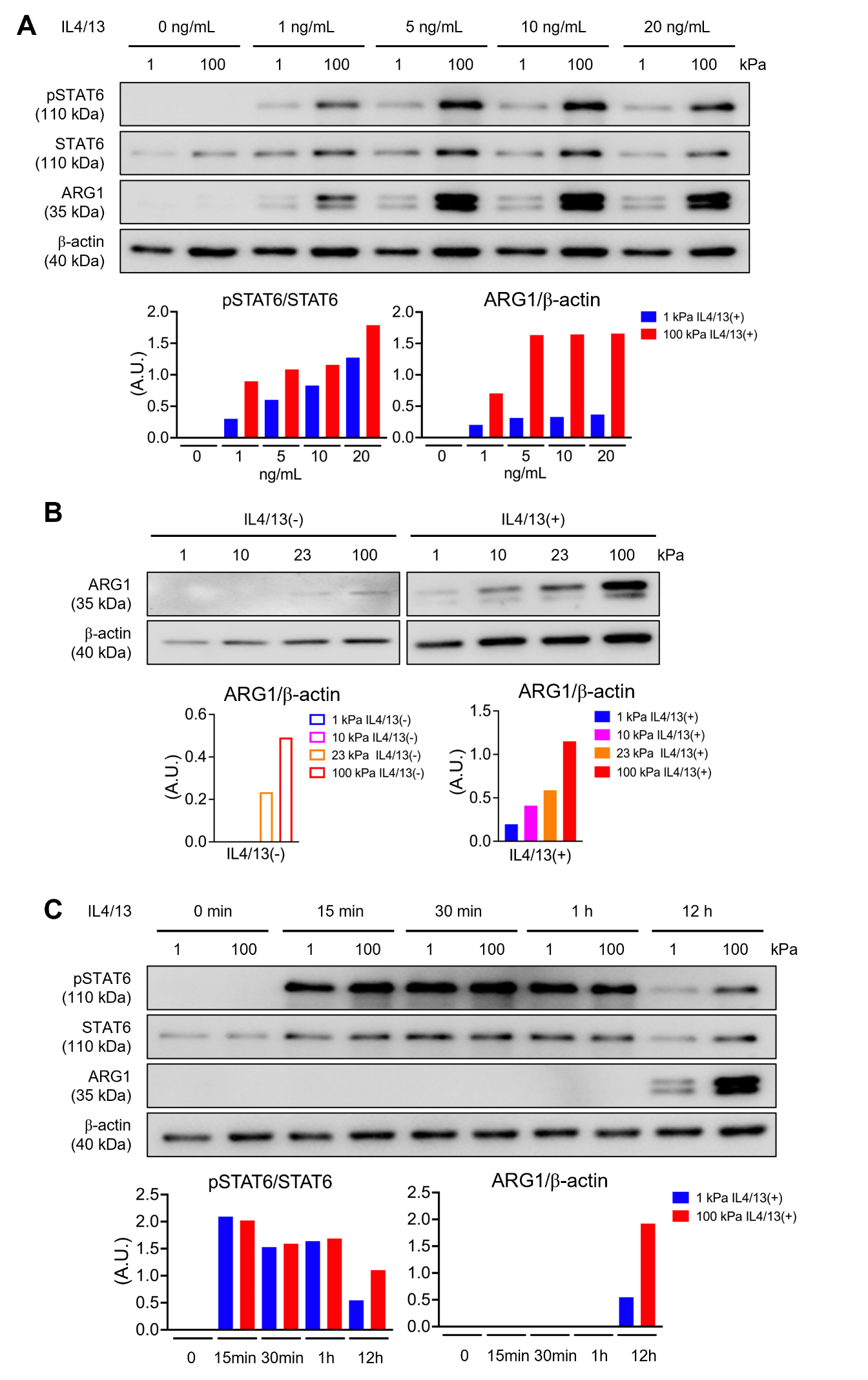


**Fig. S2. IL4/13-mediated induction of M2 macrophage markers is enhanced by elevated matrix rigidity. (A)** Western blot images showing pSTAT6, STAT6, ARG1, and β-actin in BMDMs exposed to varying concentrations of IL4/13 (0, 1, 5, 10, and 20 ng/mL) for 12 h on either 1 or 100 kPa PA gel. **(B)** Western blots of ARG1 and β-actin in BMDMs cultured on substrates with different rigidity levels (1, 10, 23, and 100 kPa) with or without IL4/13 treatment for 12 h. **(C)** BMDMs were treated with IL4/13 (20 ng/mL) for 0, 15 min, 30 min, 1 h, or 12 h on either 1 or 100 kPa PA gel. Western blot images of pSTAT6, STAT6, ARG1, and β-actin are shown. Semi-quantitative analysis of pSTAT6 and ARG1 bands was performed by normalizing to STAT6 and β-actin, respectively.


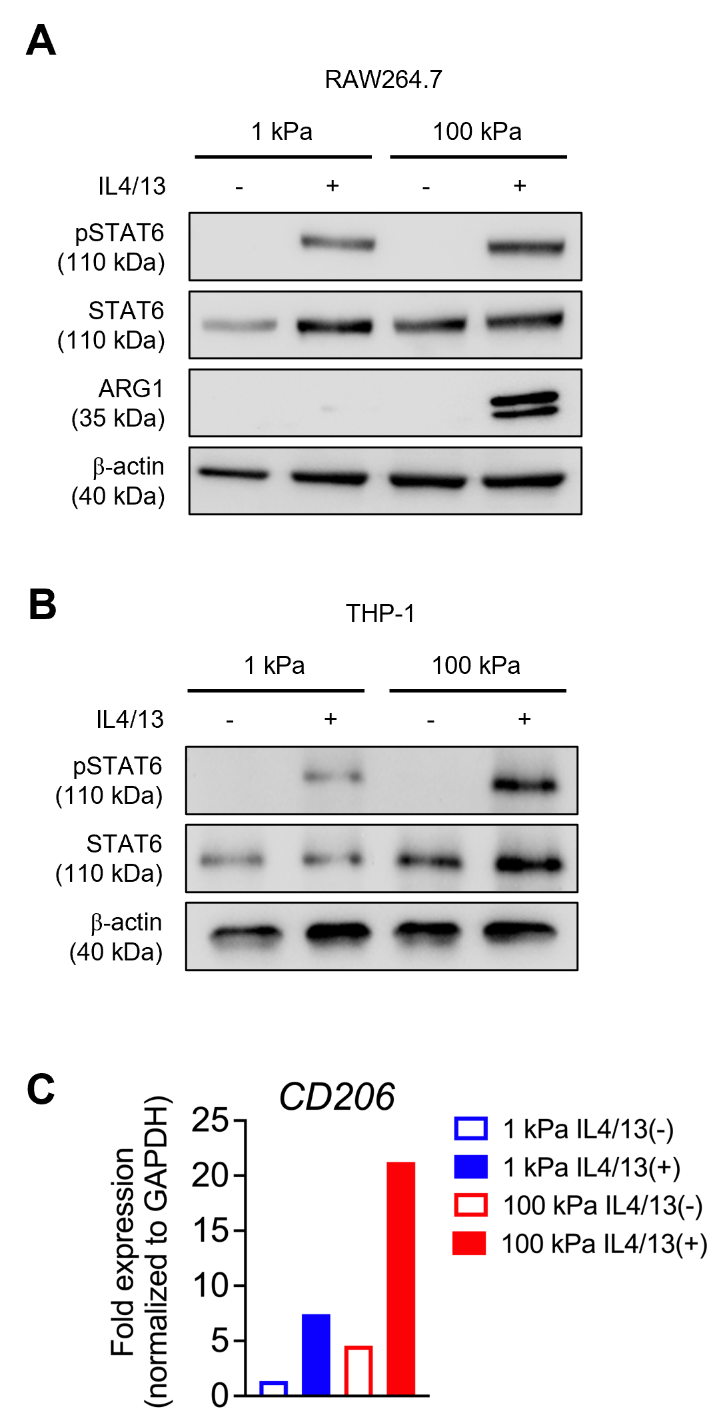


**Fig. S3. IL4/13-induced upregulation of M2 macrophage markers is enhanced in high rigidity matrices. (A, B)** Representative Western blot images of pSTAT6, STAT6, ARG1, and β-actin in RAW264.7 and THP-1 cells cultured on PA gels with rigidity levels of 1 kPa or 100 kPa, with or without IL4/13 treatment for a duration of 12 h. **(C)** qRT-PCR analysis of the expression of *CD206* in THP-1 cells*,* normalized to *GAPDH*.


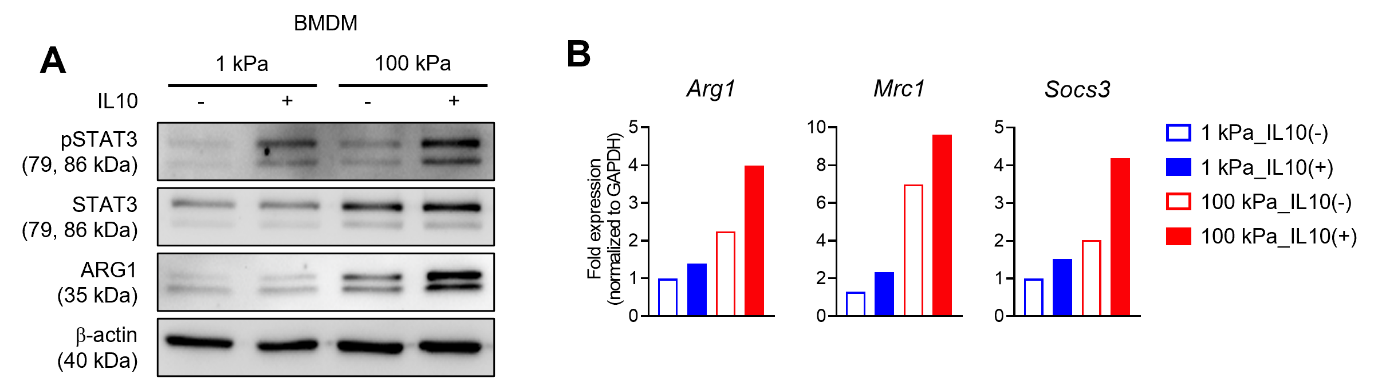


**Fig. S4. IL10-driven induction of M2 macrophage markers is augmented in response to increased matrix rigidity. (A)** Representative Western blot images of pSTAT3, STAT3, ARG1, and β-actin in BMDMs cultured on 1 kPa or 100 kPa PA gels, with or without IL10 (100 ng/mL) treatment for 12 h. **(B)** qRT-PCR analysis assessing the expression of *Arg1*, *Mrc1*, and *Socs3* normalized to *GAPDH* in these cells.


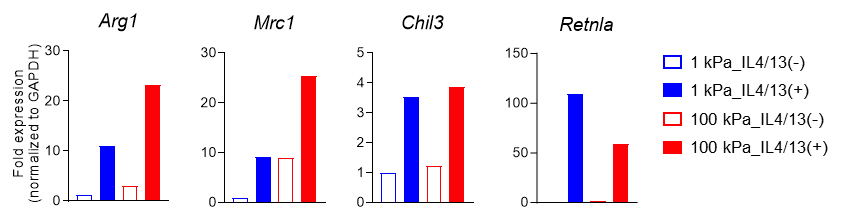


**Fig. S5. M2 macrophage-associated gene expression is synergistically regulated by cytokines and matrix rigidity.** qRT-PCR analysis for the expression of M2-associated genes (*Arg1*, *Mrc1*, *Chil3*, and *Retnla*) in BMDMs cultured on PA gels of 1 kPa or 100 kPa rigidity, with or without IL4/13 treatment.

**
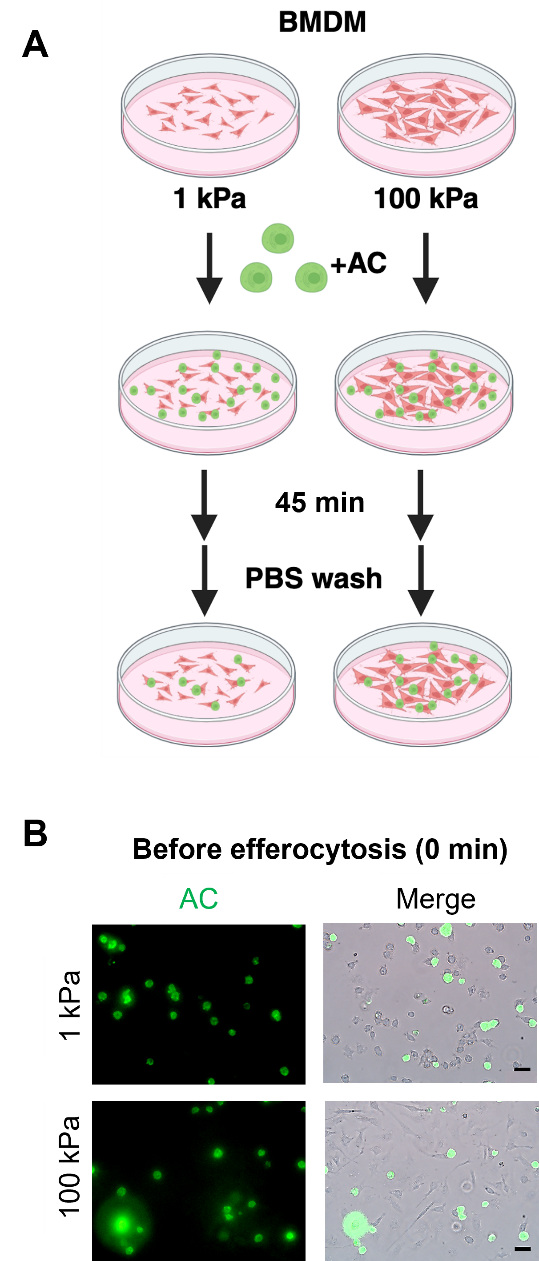
**

**Fig. S6****.** (A) Schematic illustrating the procedure of the *in vitro* efferocytosis assay. PKH67-labeled UV-irradiated apoptotic Jurkat cells (ACs) were co-cultured with BMDMs on 1 kPa or 100 kPa gels for 45 minutes. Subsequently, non-engulfed ACs were completely removed by multiple PBS washes. (B) Representative images of ACs (stained in green) and BMDMs (bright field) before efferocytosis**.**

**
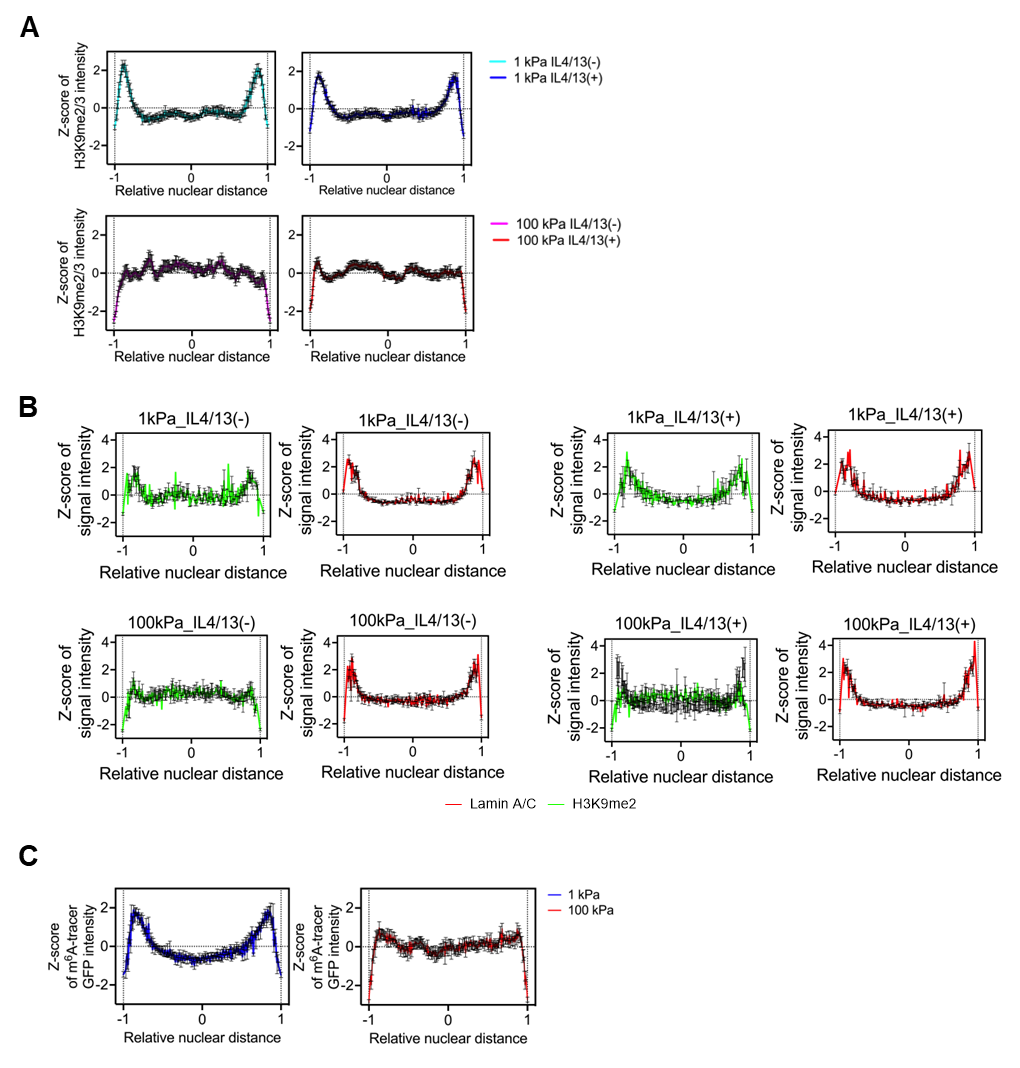
****Fig. S7. The fluorescence signal distribution graphs were replotted to show mean values with standard deviations.** (A), (B), and (C) correspond to the main figures in Fig. 2A for H3K9me2/3 immunostaining, Fig. 2D,E for co-immunostaining of H3K9me2 and Lamin A/C, and Fig. 2G for m6A tracer GFP fluorescence, respectively.

**
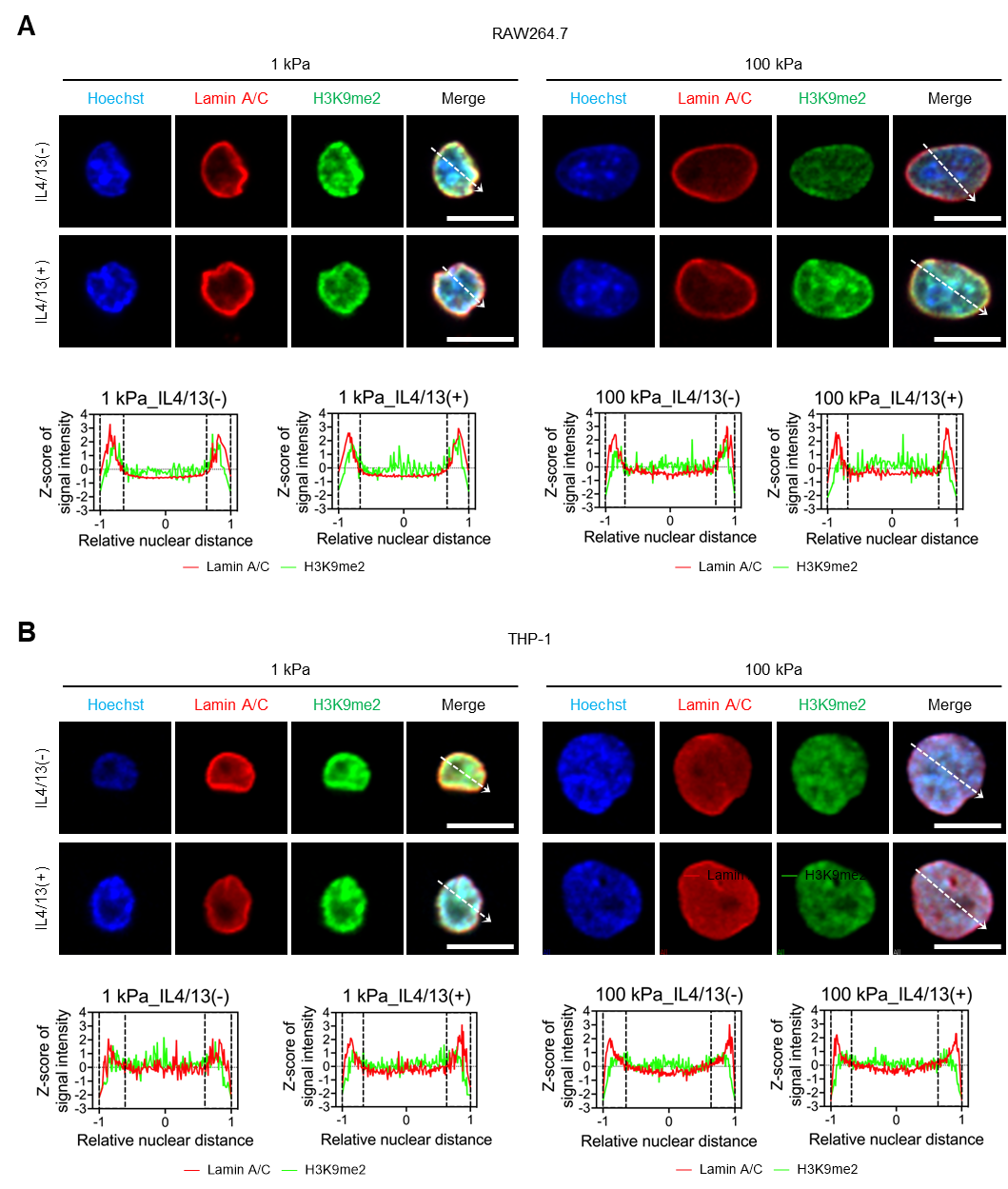
**

**Fig. S8. Laminal enrichment of heterochromatin mark, H3K9me2, is attenuated in macrophages by sensing high matrix rigidity.** (A,B) Representative co-immunostaining images exhibiting Lamin A/C (red), H3K9me2 (green), and Hoechst-33342 (blue) in RAW264.7 (A) and THP-1 cells (B), cultured on PA gels of either 1 kPa or 100 kPa rigidity, with or without IL4/13 treatment (scale bars = 10 µm). The distribution graphs of the fluorescent intensity represent the mean Z-scores of normalized fluorescence signal intensity, calculated from more than *n* = 30. Dashed arrows denote the positions of line signal intensity profiles.


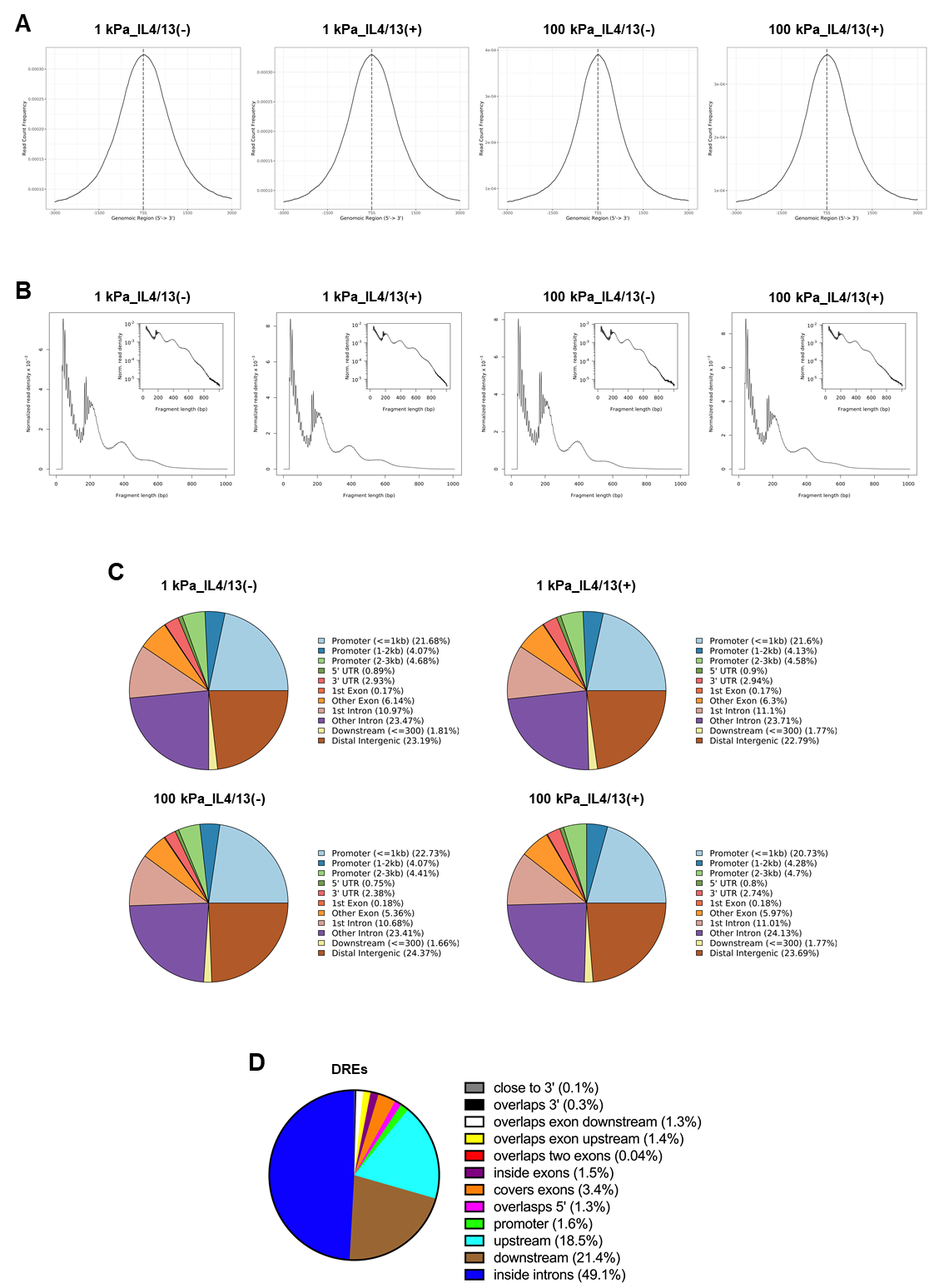


**Fig. S9. Quality assessment of ATAC-seq dataset.** **(A)** Bell-shaped transcription start site (TSS) enrichment plots showing that read counts are enriched at TSS within genomic regions encompassing ± 3 kbp from TSS in all samples. **(B)** Fragment size distribution plot showing enrichment around 100 and 200 base pair (bp), indicating nucleosome-free and mono-nucleosome-bound fragments. **(C)** Peak annotation pie charts showing that typical patterns of peak distribution in all samples. **(D)** Pie chart showing the peak distribution of distinct regulatory elements (DREs) unique in one or more of the four conditions compared to the other conditions (|fold change|≥1.5, normalized data (log2)≥1).

**
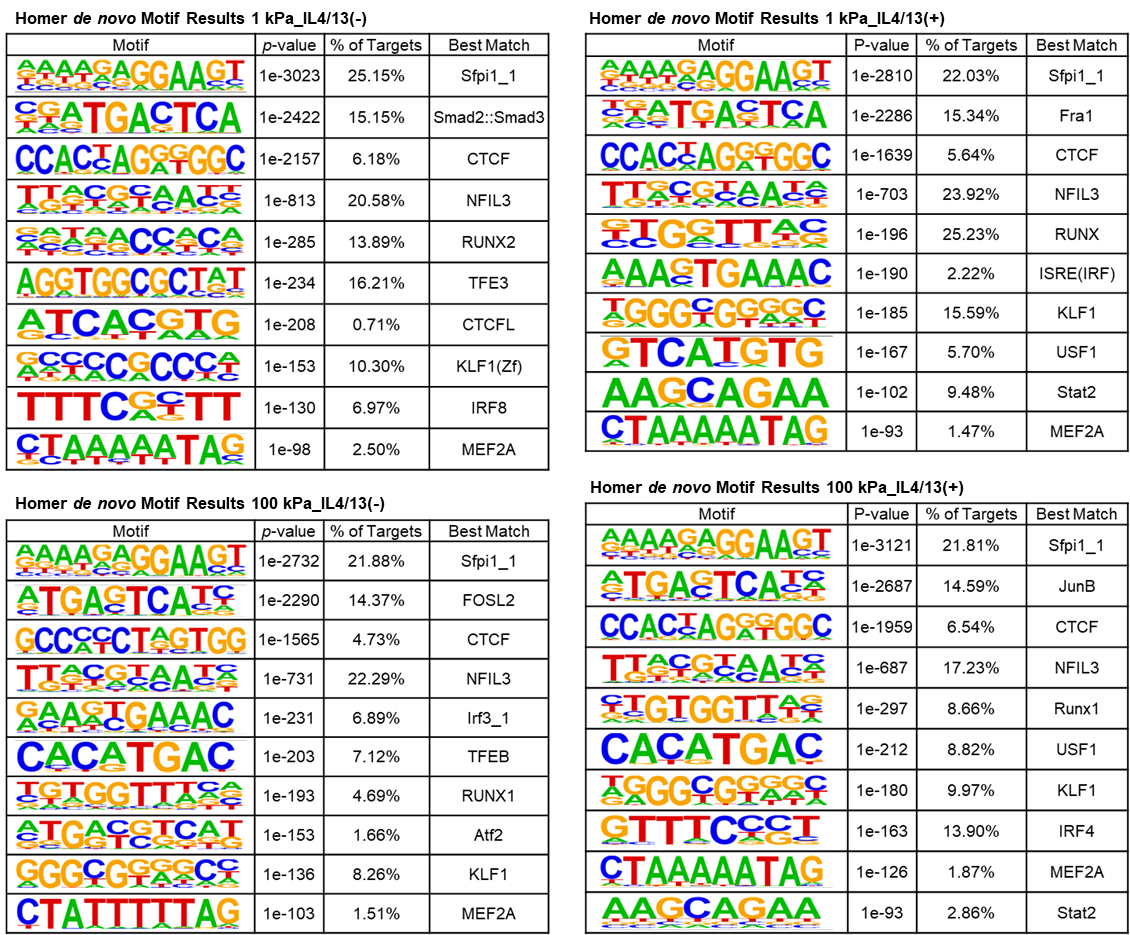
**

**Fig. S10. Identification of the top 10 novel transcription factor binding motifs through Homer software across four distinct BMDM culture conditions** (1 kPa_IL4/13(-), 1 kPa_IL4/13(+), 100 kPa_IL4/13(-), and 100 kPa_IL4/13(+)).

**
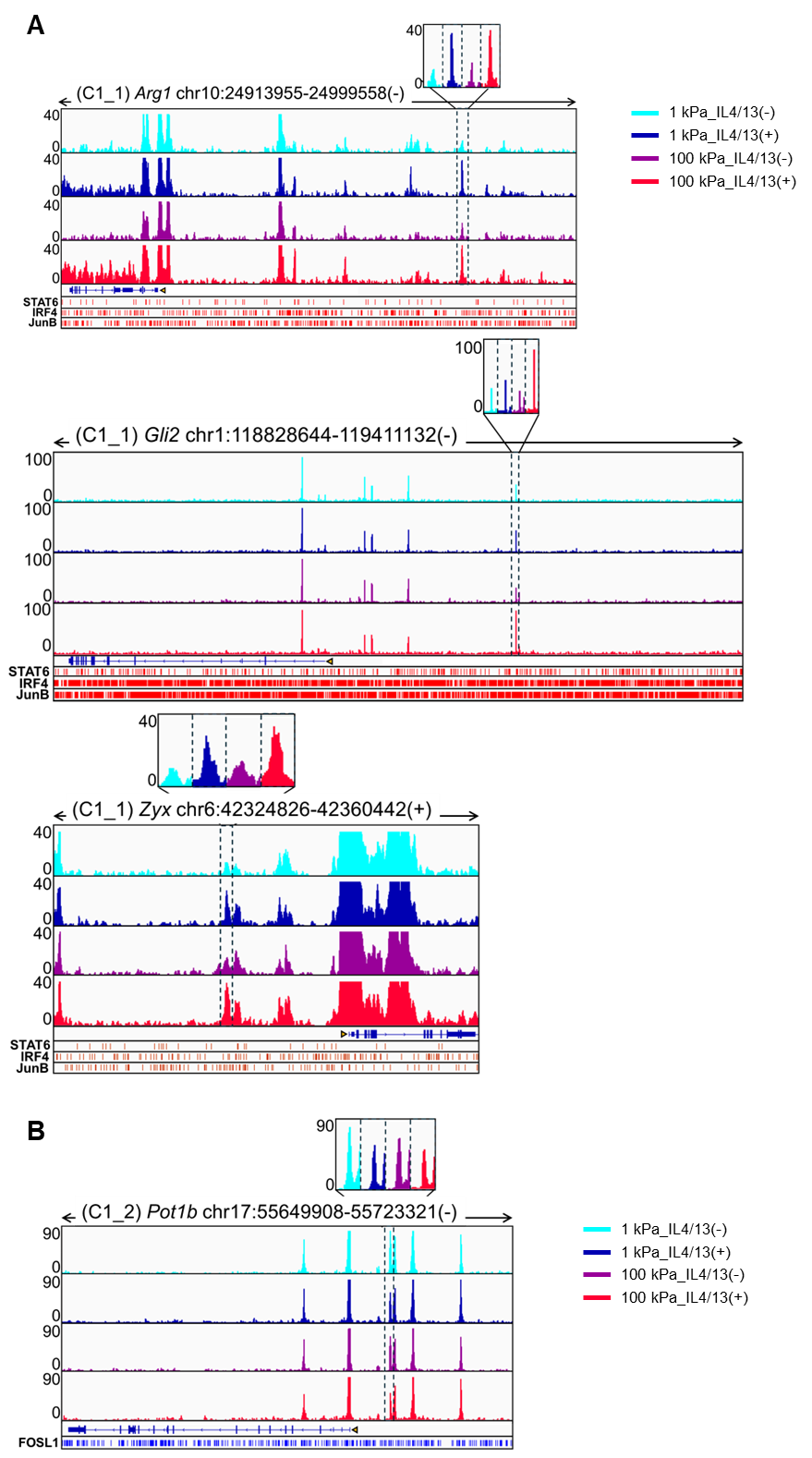
**

**Fig. S11. Normalized ATAC-seq profiles across the gene locus in C1_1 and C1_2 clusters.** **(A, B)** Visualization of normalized DRE peaks spanning the chosen gene locus within C1_1 **(A)** and C1_2 **(B)** clusters across the four distinct BMDM culture conditions. Binding sites of M2-activating TFs, including STAT6, IRF4, and JunB, are denoted by red bars, while the binding sites of an M2-inhibiting TF, FOSL1, is represented by blue bars.


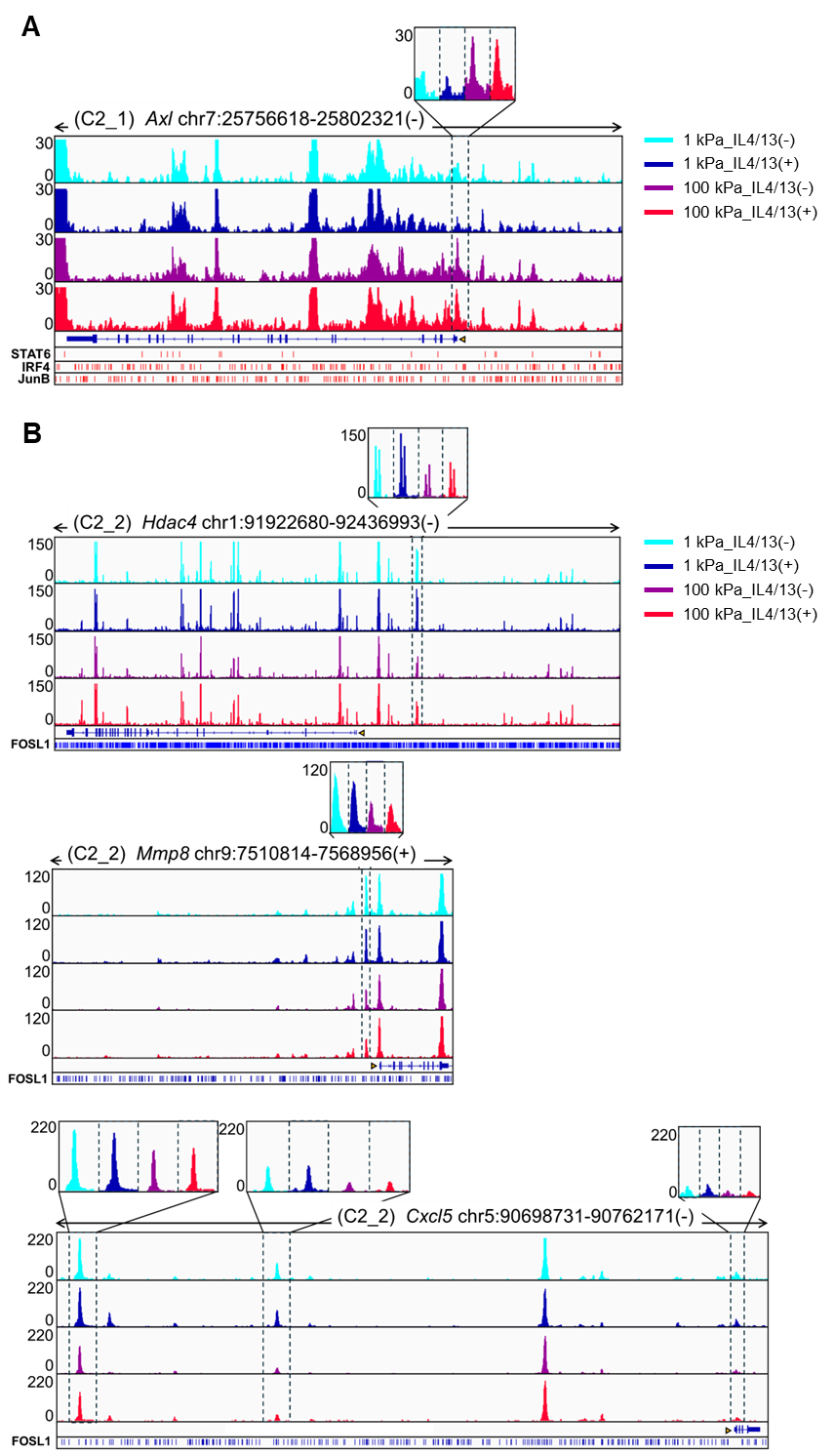


**Fig. S12. Normalized ATAC-seq profiles across the gene locus in C2_1 and C2_2 clusters.** **(A, B)** Visualization of normalized DRE peaks spanning the chosen gene locus within C2_1 **(A)** and C2_2 **(B)** clusters across the four distinct BMDM culture conditions. Binding sites of M2-activating TFs, including STAT6, IRF4, and JunB, are denoted by red bars, while the binding sites of an M2-inhibiting TF, FOSL1, is represented by blue bars.

**
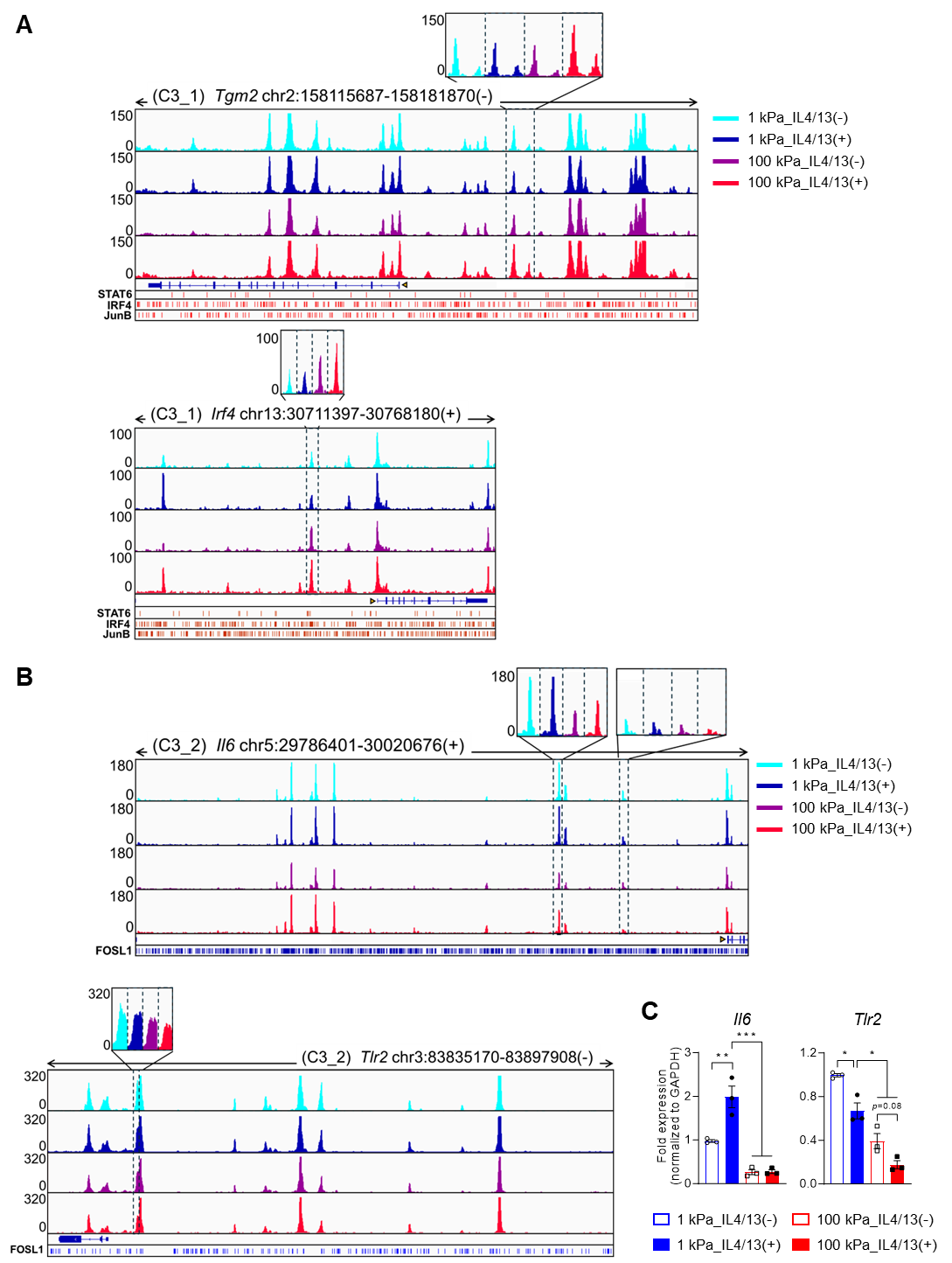
**

**Fig. S13. Normalized ATAC-seq profiles across the gene locus in C3_1 and C3_2 clusters.** **(A, B)** Visualization of normalized DRE peaks spanning the chosen gene locus within C3_1 **(A)** and C3_2 **(B)** clusters across the four distinct BMDM culture conditions. Binding sites of M2-activating TFs, including STAT6, IRF4, and JunB, are denoted by red bars, while the binding sites of an M2-inhibiting TF, FOSL1, is represented by blue bars. **(C)** qRT-PCR analysis of *Il6 and Tlr2* in BMDMs cultured on 1 kPa or 100 kPa PA gels with or without IL4/13 treatment (*n* = 3 replicates; * *p* < 0.05, ** *p* < 0.005, and *** *p* < 0.001; one-way ANOVA).

**
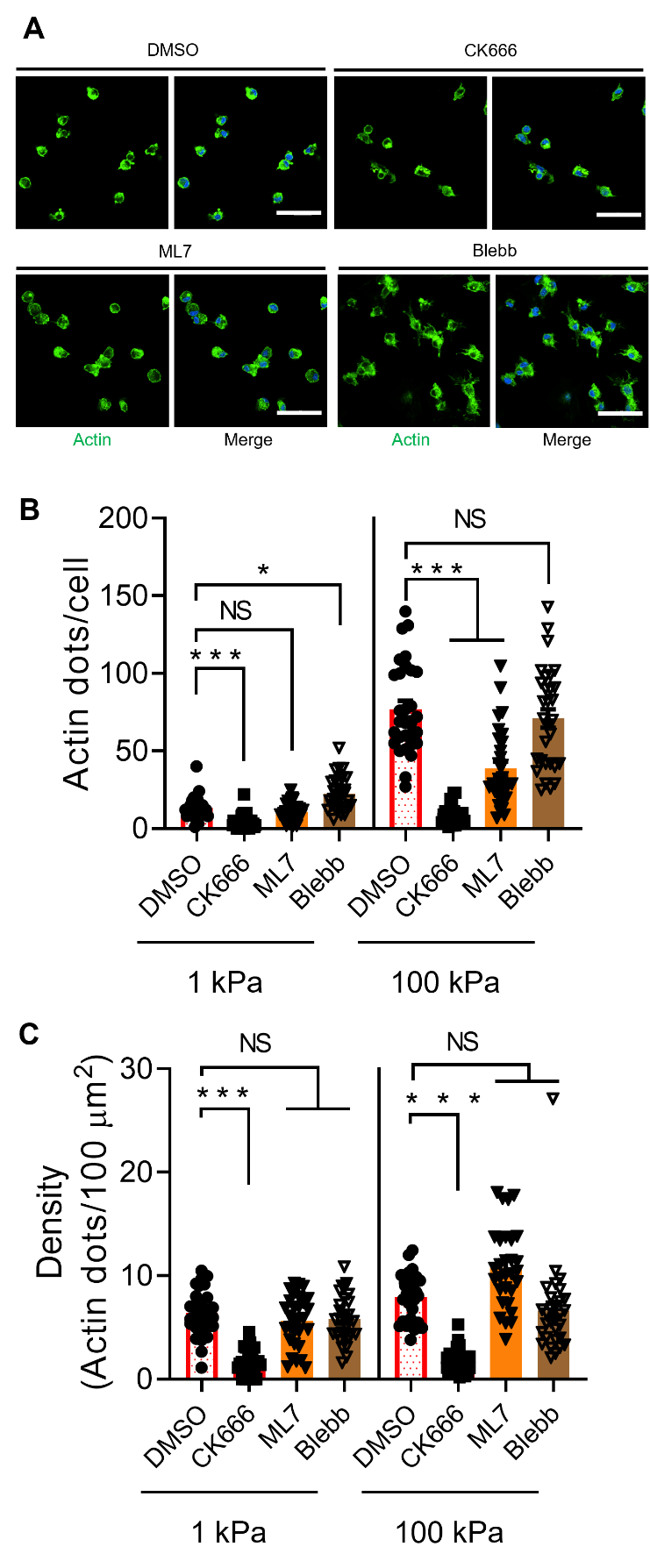
**

**Fig. S14.** (A) Representative immunostaining images of phalloidin (green) and Hoechst-33342 (blue) in BMDMs cultured on 1 kPa PA gels in the presence of IL4/13 after treatment with CK666, ML7, and blebbistatin (Blebb), or DMSO as a vehicle (scale bars = 50 µm). (B,C) The number of actin dots per cell and density (number of actin dots/100 µm^2^) on 1 kPa are quantified, compared with those on 100 kPa. Data are presented as mean ± SEM (*n* = 30 cells/group; * *p* < 0.05 and *** *p* < 0.001; one-way ANOVA). ns, not significant. Data on 100 kPa are also included for comparison .

**
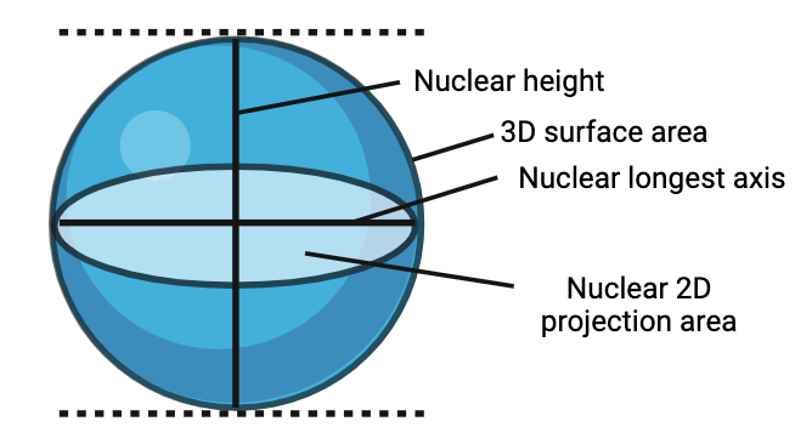
**

**Fig. S15.** Nuclear morphological parameters: Nuclear 2D projection area is calculated from a flattened nucleus of plane view. Nuclear flattening index (NFI) is calculated by dividing the length of the longest axis of a nucleus by its height, where a higher NFI value signifies greater nuclear flattening. Nuclear 3D surface area is the total area of nuclear outer surface (figure referenced in [1]).

**
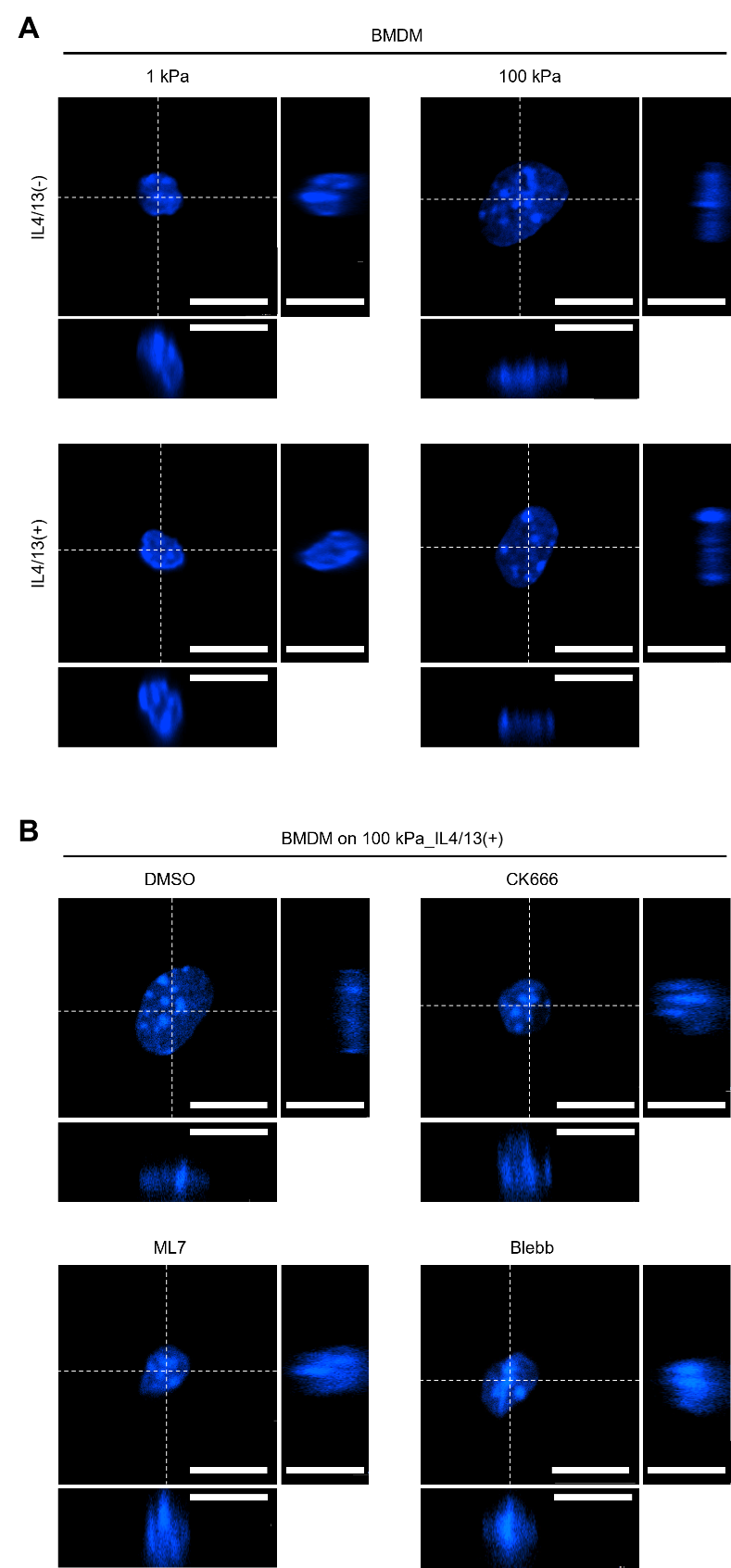
**

**Fig. S16. Nuclear morphology of BMDMs in different conditions. (A)** Representative top- and side-view images depict the distinct nuclear morphology of BMDMs cultured on either 1 kPa or 100 kPa PA gel in the absence or the presence of IL4/13 (scale bars = 10 µm). **(B)** Representative images of nuclear morphology of BMDMs cultured on the 100 kPa_IL4/13(+) condition after treatment with CK666, ML7, blebbistatin (Blebb), or DMSO as a vehicle (scale bars = 10 µm). Visualization utilized DAPI (blue) staining. The white dashed lines indicate the sectioning points of side views.

**
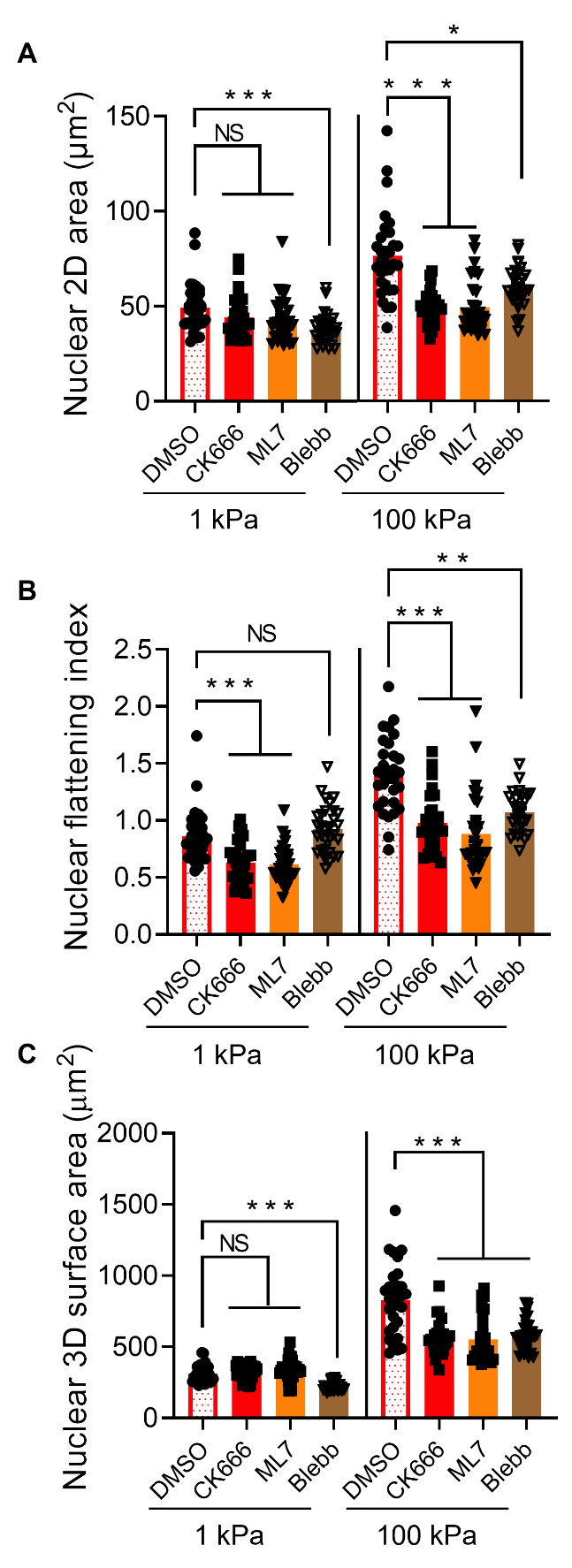
**

**Fig. S17**. Changes in (A) nuclear 2D area (µm^2^), (B) nuclear flattening index (NFI), and (C) nuclear 3D surface area upon 1 kPa with the treatment of inhibitors, compared with those upon 100 kPa. Data are presented as mean ± SEM (n = more than 30 cells/group; ** p < 0.01, *** p < 0.001, NS: not significance; One-way ANOVA or Kruskal-Wallis test).


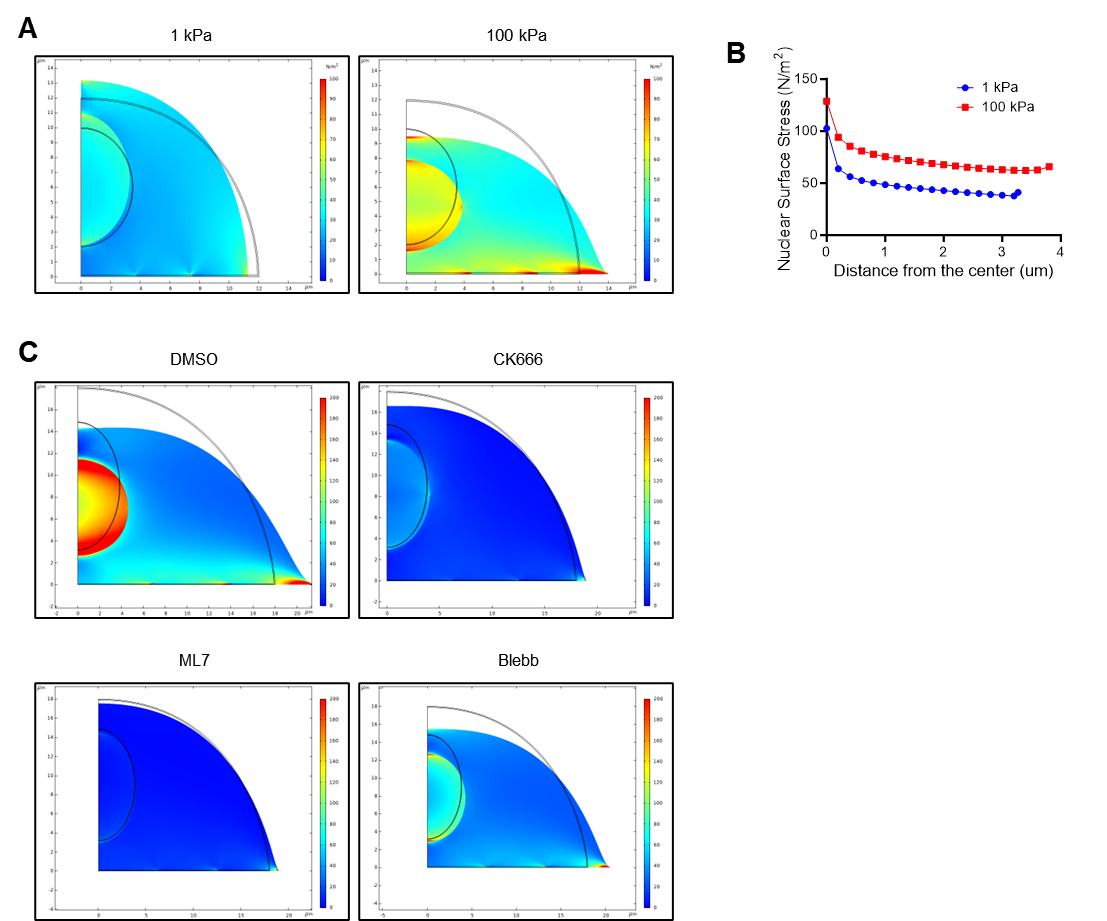


**Fig. S18. Nuclear deformation and increased nuclear surface stress under high matrix rigidity via actomyosin-mediated force transmission. (A)** 2D sectional views illustrating the deformation and stress distribution within the cell anchored to a compliant (left) or rigid (right) substrate. Cell deformation was simulated through horizontal stretching, and boundary loads were applied to the cytoplasm. The black solid curves indicate the nucleus and cell boundary before deformation, with the nucleus positioned at the center of the hemispherical cell model. Stress distribution after deformation was color-coded. **(B)** Quantification of von-Mises stress on the nuclear surface. von-Mises stresses exhibited a gradual decrease from the center to the boundary of the cell. **(C)** Deformation and stress distribution within the cells anchored to a rigid substrate after treatment with CK666, ML7, Blebbistatin (Blebb), or DMSO as a vehicle.


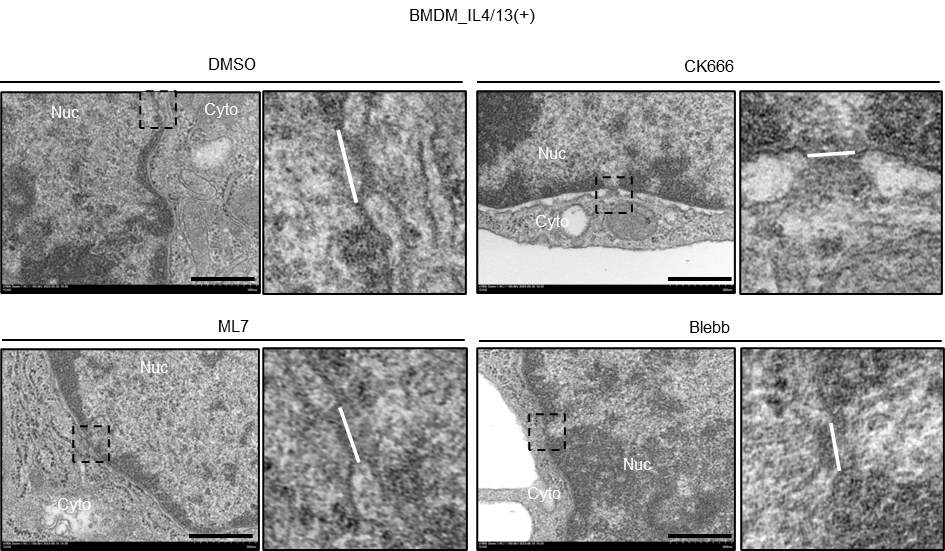


**Fig. S19. Representative TEM images of nuclear pores in actomyosin-inhibited BMDMs.** Representative TEM images of nuclear pores in BMDMs treated with DMSO, CK666, ML7, or Blebb in the presence of IL4/13 (scale bars = 100 nm). Nuclear pores within dashed boxes are captured at a higher magnification. The lengths of open nuclear pores are indicated by white lines. Nuc, nucleus; Cyto, cytoplasm.


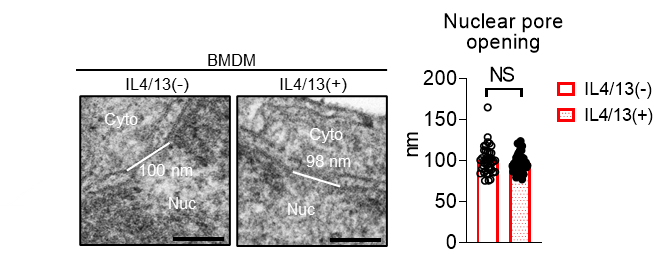


**Fig. S20. Effect of IL4/13 treatment on the nuclear pore opening** (Scale bars = 100 nm). White lines indicate the lengths of opened nuclear pores. The mean ± SEM of nuclear pore sizes was calculated from 40 pores of cells (unpaired Student *t*-test, NS: not significance).


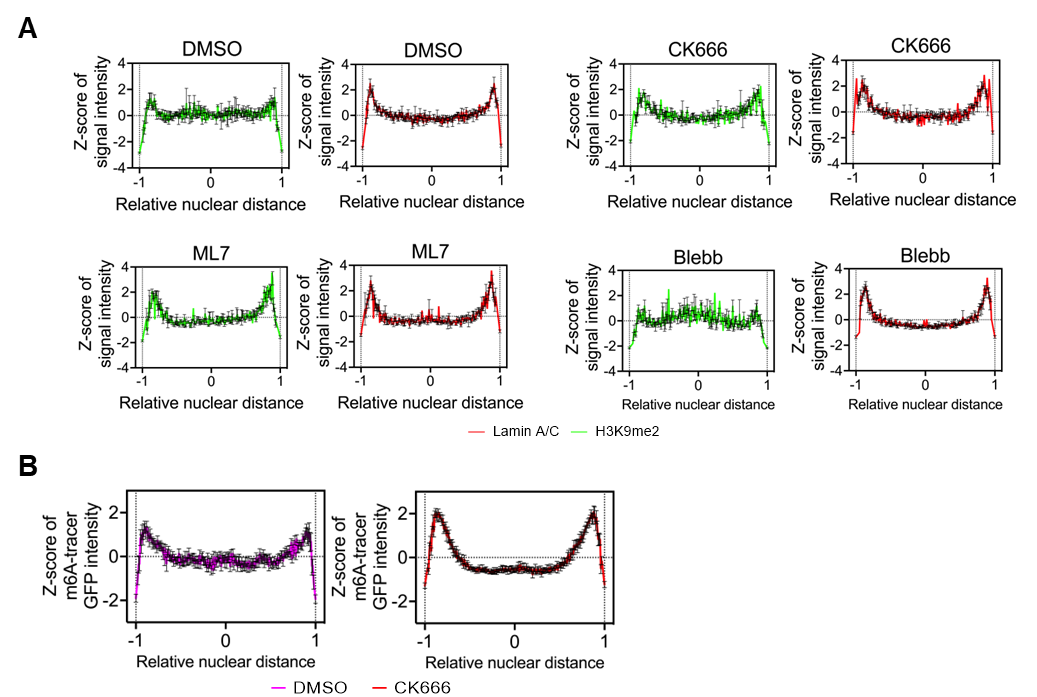


**Fig. S21. The fluorescence signal distribution graphs were replotted to show mean values with standard deviations.** (A) and (B) correspond to the main figures in Fig. 7A for co-immunostaining of Lamin A/C and H3K9me2, and Fig. 7B for m6A tracer GFP fluorescence, respectively.

**
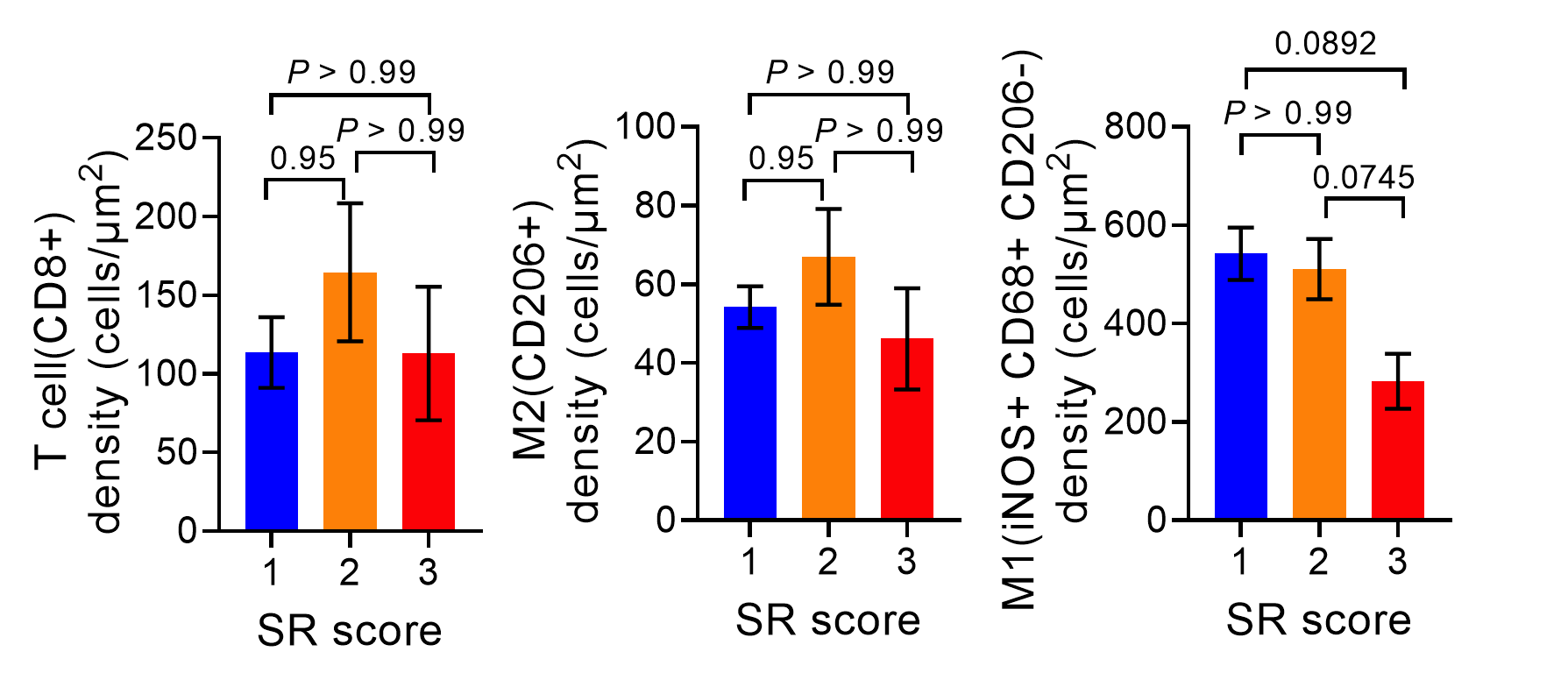
**

**Fig. S22.** The quantification graphs of cell density (cell/μm^2^) for cytotoxic T cells (CD8+), M2 macrophages (CD206+), and M1 macrophages (CD68+, iNOS+, CD206-) in tissue microarrays of human head and neck squamous cell carcinoma tissues.

**Table S1. Mechanical properties of a model cell.**

| Property | Cytosol | Nucleus | Unit | Reference |
| --- | --- | --- | --- | --- |
| Shear modulus | 50 | 80 | Pa | [2] |
| Poisson’s ratio | 0.4 | 0.4 | - | [2b, 2c, 3] |
| Density | 1 | 1.2 | kg/m^3^ | [4] |
| Young’s modulus | 200 | 300 | Pa | [5] |

**Table S2. Primer sequences used in qRT-PCR.**

| Mouse gene | Sequence |
| --- | --- |
| *mArg1* | Forward: 5’-TTATCGGAGCGCCTTTCTCA-3’ |
|  | Reverse: 5’-AGACCGTGGGTTCTTCACAA-3’ |
| *mMrc1* | Forward: 5’-TGCCAAGTGGGAAAATCTGG-3’ |
|  | Reverse: 5’-TAGGGCCACCACTGATTAGG-3’ |
| *mChil3* | Forward: 5’-GAAGCTCTCCAGAAGCAATCCT-3’ |
|  | Reverse: 5’-CTGGTAGGAAGATCCCAGCTGTA -3’ |
| *mRetnla* | Forward: 5’-CCAATCCAGCTAACTATCCCTCC-3’ |
|  | Reverse: 5’-CCAGTCAACGAGTAAGCACAG-3’ |
| *mIl10* | Forward: 5’-GTGGAGCAGGTGAAGAGTGAT-3’ |
|  | Reverse: 5’-AGTCCAGCAGACTCAATACACA-3’ |
| *mTnfα* | Forward: 5’-CCACGCTCTTCTGTCTACTG-3’ |
|  | Reverse: 5’-CTGATGAGAGGGAGGCCATT-3’ |
| *mIl6* | Forward: 5’-CTTCACAAGTCGGAGGCTTAAT-3’ |
|  | Reverse: 5’-ACTCCAGGTAGCTATGGTACTC-3’ |
| *mJak2* | Forward: 5’-TTGTGGTATTACGCCTGTGTATC-3’ |
|  | Reverse: 5’-ATGCCTGGTTGACTCGTCTAT-3’ |
| *mTgfbi* | Forward: 5’-CATTGGCACCAACAAGAAATAC-3’ |
|  | Reverse: 5’-CTTTTCATATCCAGGACAGCAC-3’ |
| *mPparg* | Forward: 5’-GGCCTCCCTGATGAATAAA-3’ |
|  | Reverse: 5’-GCTCCATAAAGTCACCAAAG-3’ |
| *mSocs3* | Forward: 5’-CCTTTGACAAGCGGACTCTC-3’ |
|  | Reverse: 5’-GCCAGCATAAAAACCCTTCA-3’ |
| *mCxcl5* | Forward: 5’-GGTCCACAGTGCCCTACG-3’ |
|  | Reverse: 5’-GCGAGTGCATTCCGCTTA-3’ |
| *mTlr2* | Forward: 5’-ACTTCTCTGCTTTTCGTTCATC-3’ |
|  | Reverse: 5’-CTCGTAGCATCCTCTGAGATTT-3’ |
| *mGAPDH* | Forward: 5’-CTGCACCACCAACTGCTTAG-3’ |
|  | Reverse: 5’-GTCTTCTGGGTGGCAGTGAT-3’ |
| *hCD206* | Forward: 5'-TCCGGGTGCTGTTCTCCTA-3' |
|  | Reverse: 5'-CCAGTCTGTTTTTGATGGCACT-3' |
| *hGAPDH* | Forward: 5’-ATGCCTCCTGCACCACCAACT-3’ |
|  | Reverse: 5’-ATGGCATGGACTGTGGTCATGAGT-3’ |

**Table S3. Antibodies used in Western blot.**

| Target antigen | Vendors or Source | Catalog number | Dilution information |
| --- | --- | --- | --- |
| Phospho-STAT6 | Cell Signaling | 56554S | 1:1000 |
| STAT6 | Cell Signaling | 5397S | 1:1000 |
| Phospho-STAT3 | Cell Signaling | 9145S | 1:1000 |
| STAT3 | Cell Signaling | 4904S | 1:1000 |
| ARG1 | Invitrogen | PA5-29645 | 1:1000 |
| H3K9me2/3 | Cell Signaling | 5327S | 1:500 |
| β-tubulin | Cell Signaling | 2146S | 1:1000 |
| β-actin conjugated with peroxidase | Cell Signaling | A3854 | 1:10000 |

**Table S4. Antibodies used in immunofluorescent staining.**

| Target antigen | Vendors or Source | Catalog number | Dilution information |
| --- | --- | --- | --- |
| Phospho-STAT6 | Cell Signaling | 9361S | 1:400 |
| Lamin A/C | Cell Signaling | 4777S | 1:200 |
| Lamin B1 | Abcam | Ab16048 | 1:200 |
| VINCULIN | Abcam | ab129002 | 1:1000 |
| H3K9me2/3 | Cell Signaling | 5327S | 1:200 |
| H3K9me2 | Invitrogen | PA5-16195 | 1:200 |

**References**

[1] J. K. Kim, S. B. Han, S. I. Park, I. S. Kim, D. H. Kim, *Biomaterials* **2022**, *290*, 121859, <https://doi.org/10.1016/j.biomaterials.2022.121859>.

[2] a) S. Yamada, D. Wirtz, S. C. Kuo, *Biophys J* **2000**, *78* (4), 1736, <https://doi.org/10.1016/S0006-3495(00)76725-7>; b) P. Panorchan, B. W. Schafer, D. Wirtz, Y. Tseng, *J Biol Chem* **2004**, *279* (42), 43462, <https://doi.org/10.1074/jbc.M402474200>; c) J. K. Kim, A. Louhghalam, G. Lee, B. W. Schafer, D. Wirtz, D. H. Kim, *Nat Commun* **2017**, *8* (1), 2123, <https://doi.org/10.1038/s41467-017-02217-5>.

[3] a) A. Vaziri, H. Lee, M. R. K. Mofrad, *Journal of Materials Research* **2006**, *21* (8), 2126, <https://doi.org/10.1557/jmr.2006.0262>; b) C. R. Ethier, C. A. Simmons, *Introductory biomechanics : from cells to organisms*, Cambridge University Press, Cambridge **2008**.

[4] a) S. Heyden, M. Ortiz, *Computer Methods in Applied Mechanics and Engineering* **2017**, *314*, 314, <https://doi.org/10.1016/j.cma.2016.08.026>; b) U. Moran, R. Phillips, R. Milo, *Cell* **2010**, *141* (7), 1262, <https://doi.org/10.1016/j.cell.2010.06.019>.

[5] E. M. L. L.D. Landau, *Theory of Elasticity*, Pregamon Press, **1970**.
